# Decreased mitochondrial transcription factor A and mitochondrial DNA copy number promote cyclin-dependent kinase inhibitor 1A expression and reduce tumorigenic properties of colorectal cancer cells

**DOI:** 10.1007/s12672-024-01538-4

**Published:** 2024-11-24

**Authors:** Jessika Buchwaldt, Tania Fritsch, Monika Hartmann, Hagen Roland Witzel, Michael Kloth, Wilfried Roth, Katrin E. Tagscherer, Nils Hartmann

**Affiliations:** 1 Institute of Pathology, University Medical Center, Johannes Gutenberg University Mainz, Langenbeckstr. 1, 55131 Mainz, Germany; 2 Department of Medicine III, University Medical Center, Johannes Gutenberg University Mainz, Langenbeckstr. 1, 55131 Mainz, Germany

**Keywords:** Mitochondrial DNA (mtDNA), Transcription factor A mitochondrial (TFAM), Cyclin-dependent kinase inhibitor 1A (CDKN1A/p21), Mitochondria, Cell proliferation, Senescence, Colon cancer

## Abstract

**Purpose:**

Colorectal cancer is one of the most common and deadliest cancer types worldwide. In the last years, changes in the mitochondrial DNA (mtDNA) copy number have been described to correlate with the prognostic outcome for colorectal cancer patients by impacting different tumorigenic properties. One key regulator of mtDNA is the mitochondrial transcription factor A (TFAM) that acts as a limiting factor of mtDNA copy number. Here, we investigated the effect of *TFAM* deficiency on mtDNA and tumorigenic properties in the human colorectal cancer cell line SW480.

**Methods:**

*TFAM* expression was stably downregulated in the colorectal cancer cell line SW480 using the CRISPR-Cas9 approach. To dissect the molecular alterations induced by deletion of *TFAM*, RNA sequencing and gene set enrichment analysis was performed on *TFAM*-wild-type and *TFAM*-deficient SW480 cells. Functional consequences of *TFAM* downregulation were assessed in cellular assays.

**Results:**

We showed that *TFAM* deficiency leads to decreased mtDNA copy number and reduced expression of mtDNA-encoded genes. *TFAM*-deficient cells also revealed higher activity of senescence-associated β-galactosidase and decreased cell growth parameters. Moreover, RNA sequencing showed that the expression of cyclin dependent kinase inhibitor 1A (*CDKN1A/p21*) is significantly increased in *TFAM*-deficient cells.

**Conclusion:**

Our results suggest that TFAM-induced changes of the mitochondrial genome lead to upregulated *CDKN1A/p21* expression in colorectal cancer cells identifying p21 as a new possible linker between mitochondria and nucleus.

**Supplementary Information:**

The online version contains supplementary material available at 10.1007/s12672-024-01538-4.

## Introduction

Colorectal cancer is the third most common tumor entity and the second leading cause of cancer-related death worldwide [1]. The 5-year-survival rate of patients with diagnosed colorectal cancer is between 50 and 60%, with higher rates in the initial stages of tumors (75–90%) compared to advanced stages (≤ 15%) [2, 3]. Given that most tumor cases are diagnosed at a late stage following metastasis, an early diagnosis and prognosis are crucial for the survival of patients. It has been shown that metastatic progression in colorectal cancer is promoted by changes in the expression of mitochondria-related genes and changes in the mitochondrial DNA (mtDNA) copy number [4]. In particular, high mtDNA content in tumor tissues was associated with larger tumor size, advanced TNM stage, higher serum level of carcinoembryonic antigen, vascular emboli, and liver metastases [5]. Furthermore, high mitochondrial DNA (mtDNA) content in tumor tissue predicted poor outcome and a higher risk of metastases after surgery [5].

The mtDNA is a double-stranded, circular genome that harbors 37 genes [6]. It encodes for 13 subunits of complexes of the oxidative phosphorylation system (OXPHOS) and therefore has an impact on mitochondrial respiration [7]. Moreover, the mtDNA encodes for two ribosomal RNAs and 22 transfer RNAs that are necessary for the synthesis of these subunits [7]. The replication and transcription of the genome is driven by two non-coding regions that are called control regions or displacement loops [7]. Even though they are located on the mtDNA itself, the expression of the genome still highly depends on nuclear-encoded proteins.

The mitochondrial transcription factor A (TFAM) is one of the nuclear-encoded proteins that plays an important role for mtDNA genomic integrity and expression. The protein binds to mtDNA through high mobility group (HMG) box motifs as a homodimer and is part of the transcription initiation complex in mitochondria [8]. TFAM fully coats the mtDNA and is responsible for packaging the genome into mitochondrial nucleoids [9]. Moreover, TFAM levels correlate with mtDNA content in transgenic mice and zebrafish embryos suggesting that TFAM can function as a limiting determinant for mtDNA copy number [10, 11]. These limitations in copy number have been associated with OXPHOS deficiency and changes in the expression of respiratory chain complexes underlining that TFAM has an impact on mitochondrial respiration [11]. Since TFAM affects the energy levels in cells, it is not surprising that the protein has been linked to changes in the tumorigenesis of cancer cells. In non-small cell lung cancer and gastric cancer cells, the depletion of *TFAM* resulted in cell cycle arrest, morphology changes and reduced proliferation [12, 13]. Furthermore, *TFAM* expression has been associated with a negative outcome for patients with pancreatic and non-small cell lung cancer through the inhibition of apoptosis [12, 14]. However, the role of TFAM in the tumorigenesis of colorectal cancer cells remains unknown.

In this study, we examined the impact of TFAM on mtDNA content and cellular signaling by comparing wild-type (wt) and *TFAM*-deficient SW480 cells. We found that *TFAM* deficiency resulted in a reduction of mtDNA copy number and changes in mtDNA-encoded proteins. Moreover, tumorigenic properties of the cells were significantly decreased after knockdown of the gene. We identified *CDKN1A/p21* expression to be regulated by TFAM and in this context found that senescence-associated β-galactosidase activity was increased in *TFAM*-deficient cells.

## Materials and methods

### Cell line and cell culture

The human colorectal cancer cell line SW480 was purchased from the American Type Culture Collection (ATCC, USA). The cells were *cultured in RPMI-1640 medium* (Thermo Fisher Scientific, Waltham, USA) supplemented with 10% fetal bovine serum (FBS, Sigma-Aldrich, St. Louis, USA) and antibiotics (100 units/ml penicillin and 100 µg/ml streptomycin). The cells were maintained in a humidified incubator with 5% CO_2_ at 37 °C. All experiments were performed with mycoplasma-free cells. Authentication of the used cell line was performed by NGS analysis confirming the SW480-specific mutations in the genes *TP53*, *KRAS* and *IDH1* (Supplementary Table S4) [15].

### Genetic manipulation of TFAM expression

*TFAM* expression was stably downregulated in the cells using CRISPR-Cas9 as previously described [16]. The used plasmid pSpCas9(BB)-2A-GFP (PX458) was a gift from Feng Zhang (Addgene plasmid #48,138). Sequences of primers for targeting exon 2 of the *TFAM* gene: 5′-TAAAGCTCAGAACCCAGGTA-3′ (forward) and 5′- TACCTGGGTTCTGAGCTTTA-3′ (reverse). For transfection, 6 × 10^5^ cells were seeded on 6-well plates and grown until 60–80% confluency. Transfection was performed using Lipofectamine 2000 according to the manufacturer’s instructions (Thermo Fisher Scientific, Waltham, USA). Cells were transfected with 4 µg plasmid DNA. GFP-positive single cell clones were selected by flow cytometry in collaboration with Array Core Facility of the University Medical Center Mainz, Germany.

### Immunoblot analysis

Cells were washed with ice-cold PBS and then lysed with cell lysis buffer (Cell Signaling Technology, Frankfurt/Main, Germany) supplemented with 2% Protease/Phosphatase Inhibitor (Cell Signaling Technology, Frankfurt/Main, Germany). Cell lysates were incubated for 15 min on ice and centrifuged at 12,700 rpm for 20 min at 4 °C. Protein concentration was measured by Bradford Assay (BioRad, Munich, Germany) and samples were prepared by adding 20–30 µg protein to 6 × buffer (350 mM Tris–HCl pH 6.8; 10.28% (w/v) SDS; 36% (v/v) glycerin; 600 mM dithiothreitol; bromphenol blue) followed by incubation for 3 min at 95 °C. Proteins were separated on 10–15% polyacrylamide gels by using SDS-PAGE and transferred to nitrocellulose membranes (BioRad, Munich, Germany). The membrane was incubated with Ponceau S staining solution (0.5% (w/v) Ponceau S (Carl Roth, Karlsruhe, Germany) and 1% (v/v) acetic acid (Thermo Fisher Scientific, Waltham, USA)) for 5 min and was cut according to the expected protein sizes. Blocking was performed for 1 h at room temperature using 5% low-fat milk powder in TBS containing 0.1% Tween 20 solution. Membranes were incubated with primary antibody overnight at 4 °C followed by HRP-labeled secondary antibody (1:3000 dilution) for 1 h at room temperature. Information about the used primary and secondary antibodies are listed in the supplementary Table S1. Visualization was performed by using Luminol-coumaric acid-solution (10 ml 100 M Tris–HCl [pH 8.5], 50 µl luminol solved in 250 mM DMSO, 25 µl courmaric acid solved in 90 mM DMSO, 4 µl H_2_O_2_ (30%)) and the chemiluminescence detection system Evolution-Capt (Vilber Lourmat, Eberhardzell, Germany).

### Analysis of CRISPR-Cas9 mediated mutations by Sanger sequencing

Genomic DNA from cells was extracted using the QIAmp^®^DNA Mini Kit according to the manufacturer’s instructions (Qiagen, Hilden, Germany). Target regions were amplified using S7 Fusion Polymerase (Biozym Scientific GmbH, Hessisch Oldendorf, Germany) and the subsequent primers: *TFAM* forward 5′- agc tca gaa ccc aga tgc aa-3′, *TFAM* reverse 5′- tat ata cct gcc act ccg cc-3′. Amplified fragments were resolved on 1% agarose gels and purified using Qiagen gel extraction kit (Qiagen, Hilden, Germany). Subsequently, the purified PCR products were cloned into pJET1.2/Blunt vector system (Thermo Fisher Scientific, Waltham, USA). The ligated constructs were transformed into *E. coli* competent bacteria (Thermo Fisher Scientific, Waltham, USA). Plasmid DNA was extracted using QIAprep Spin Miniprep Kit (Qiagen, Hilden, Germany). Sanger sequencing was performed by the StarSeq GmbH Mainz, Germany. Analysis of the sequences was done using Clustal Omega [17] and Clone Manager (Sci Ed Software, Westminster, Colorado, USA).

### Quantitative PCR (qPCR)

Total RNA was extracted from cells using the RNeasy^®^ Plus Mini Kit (Qiagen, Hilden, Germany) according to the manufacturer’s instructions. For complementary DNA (cDNA) synthesis, 1.1 µg RNA were mixed with 3 µl random primer (Promega, Walldorf, Germany) and incubated for 5 min at 70 °C. The mix was immediately transferred on ice for 30 s and 11 µl Master Mix containing 5 µl 5 × buffer (Promega, Walldorf, Germany), 0.8 µl RNAsin plus (Promega, Walldorf, Germany), 1 µl M-MLV Reverse Transcriptase (Promega, Walldorf, Germany) and 4.2 µl of 2.5 mM dNTPs (Thermo Fisher Scientific, Waltham, USA) was added. The mix was incubated for 60 min at 40 °C and the generated cDNA was used as template for qPCR. PCR amplification was performed using 2.5 ng/µl cDNA/10 µl reaction volume and SYBR Green (BioRad, Hercules, USA) according to the manufacturer´s protocol. Primer sequences and concentrations are listed in supplementary Table S2. The following parameters were used for thermal cycling: 50 °C for 2 min, 95 °C for 10 min, then 40 cycles of denaturation at 95 °C for 15 s and extension at 60 °C for 1 min followed by a melt curve stage at 95 °C for 15 s and 60 °C for 1 min. The threshold cycle number (Ct) was recorded and normalized to the *Glyceraldehyde 3-phosphate dehydrogenase* (*GAPDH*) value. Data analysis of the qPCR was performed using the “2^−ΔΔCt^-Method” as described previously [18]. Each sample was measured in triplicates in at least three independent experiments. To measure PCR efficiency, a 1 to 5 dilution series was assayed for every primer starting with a concentration of 2.5 ng/µl and ending at 0.02 ng/µl.

### Determination of mtDNA copy number

Mitochondrial DNA (mtDNA) copy number was determined using a qPCR assay. The Ct-values of two mitochondrial-specific (*NADH dehydrogenase 1* (*ND1*), *mtDLoop*) and two nuclear-specific (*GAPDH*, *Beta-2-Microglobulin* (*B2M*)) targets were analyzed in triplicates for each sample. The mtDNA copy number was calculated by the formula 2^ΔCt^, with ΔCt being the difference between the mean Ct-values of nuclear- and mitochondrial-specific targets (ΔCt = Ct_nuclear DNA_–Ct_mtDNA_ = mean Ct of GAPDH and B2M—mean Ct of ND1 and mtDLoop). Primer sequences are listed in supplementary Table S3.

### Cellular assays

The crystal violet assay was performed for determining cell viability using 96-well plates. Cells were seeded at three different cell densities and cultured for indicated times: 15 × 10^3^ cells/well for 48 h, 12.5 × 10^3^ cells/well for 72 h and 10 × 10^3^ cells/well for 96 h. Cells were washed with PBS and fixed with ethanol/methanol (1:2) mixture. After a second wash with PBS the cells were stained for 30 min with 0.01% crystal violet in water with gentle agitation at 175 rpm. After final wash with water the plates were dried for 24 h at room temperature. The crystal violet was solubilized using 33% acetic acid and photometrically quantified at 600 nm with the Tecan Spark 10 M (Tecan, Männedorf, Switzerland). To measure how cells perform induced gap closures (scratch assay), 0.05 × 10^6^ cells were seeded in 24-well culture plates in *RPMI-1640 medium* (Thermo Fisher Scientific, Waltham, USA) supplemented with 10% fetal bovine serum (FBS, Sigma-Aldrich, St. Louis, USA) and antibiotics (100 units/ml penicillin and 100 µg/ml streptomycin). Scratches were generated in confluent cell monolayers with a 200 µl-pipette tip and cell debris were removed by washing with PBS. Afterwards, the scratch closure was measured after 48 and 72 h. The cells were photographed using the Eclipse TS100-F microscope (Nikon, Tokio, Japan) and the cell-free gap was measured using ImageJ [19]. For determining ROS production, cells were incubated with the fluorescent H2DCF-DA (2,7-dichlorodihydrofluorescein diacetate; 5 μM; Biozol, Eching, Germany) for 30 min at 37 °C. Cells were subjected to flow cytometry analysis using a Becton–Dickinson FACScalibur cytometer and Cell Quest Software. Activity of beta-galactosidase was measured using the mammalian beta-galactosidase assay kit (Thermo Fisher Scientific, Waltham, USA) according to the manufacturer’s instructions. Photometrical quantification at 405 nm was performed with the Tecan Spark 10 M (Tecan, Männedorf, Switzerland) and beta-galactosidase activity of control cells was normalized to one.

### RNA-sequencing

Total cell RNA was isolated using the RNeasy^®^ Plus Mini Kit (Qiagen, Hilden, Germany) per manufacturer’s instructions and transcribed into cDNA using the NEB standard protocol for cDNA synthesis with the ProtoScript^®^ II Reverse Transcriptase (New England Biolabs, Frankfurt/Main, Germany). RNA-Sequencing was performed for all selected cell clones (TFAM-KD #1, TFAM-KD #2, TFAM-KD #3, TFAM-KD #4) using the AmpliSeq™ Transcriptome Human Gene Expression Panel (Illumina, San Diego, CA, USA) and the AmpliSeq™ Library PLUS Kit (Illumina, San Diego, CA, USA) according to the manufacturer’s instructions. For library sequencing the NextSeq500 System (Illumina, San Diego, CA, USA) was used with single-read sequencing and 76 bp reads. The sequencing depth was 20 million reads per sample. Generated data were quantified using the Salomon tool [20] and differential expression analysis was performed with the Bioconductor package DESeq2 for R according to the DESeq2 manual [21]. Genes with Benjamin Hochberg adjusted *p*-value (padj) ≤ 0.05 were considered significant. Gene set enrichment analysis (GSEA) was performed using GSEA software provided by Broad Institutes (http://www.broad.mit.edu/gsea/) accessed on 28 January 2024 [22, 23]. The normalized enrichment score (NES) reflected the degree of overrepresentation for each group at the peak of the entire set. The analysis was performed using classic enrichment statistic and gene set based permutation test with 1000 permutations. “Ratio of classes” was selected as metric for ranking genes. Gene sets with NOM p-value < 0.01 and false discovery rate (FDR) < 0.25 were considered significantly enriched in a priori defined set of genes.

### Statistical analysis

All statistical analyses were performed using Microsoft Excel (Microsoft Corporation, Redmond, WA, USA) and RStudio (Version 4.3.2). Three experiments were compared in each case. All p-values ≤ 0.05 were considered significant: *p ≤ 0.05, **p ≤ 0.01, and ***p ≤ 0.001. Data were visualized using Microsoft Excel and Inkscape [24]. Differential expression analysis and statistical testing of the RNA-sequencing data was performed in RStudio using the Bioconductor package DESeq2 [21] according to https://genviz.org/ and the Salmon tool [20].

## Results

### Stable downregulation of TFAM expression in colorectal cancer cells using CRISPR-Cas9

**Fig. 1:**
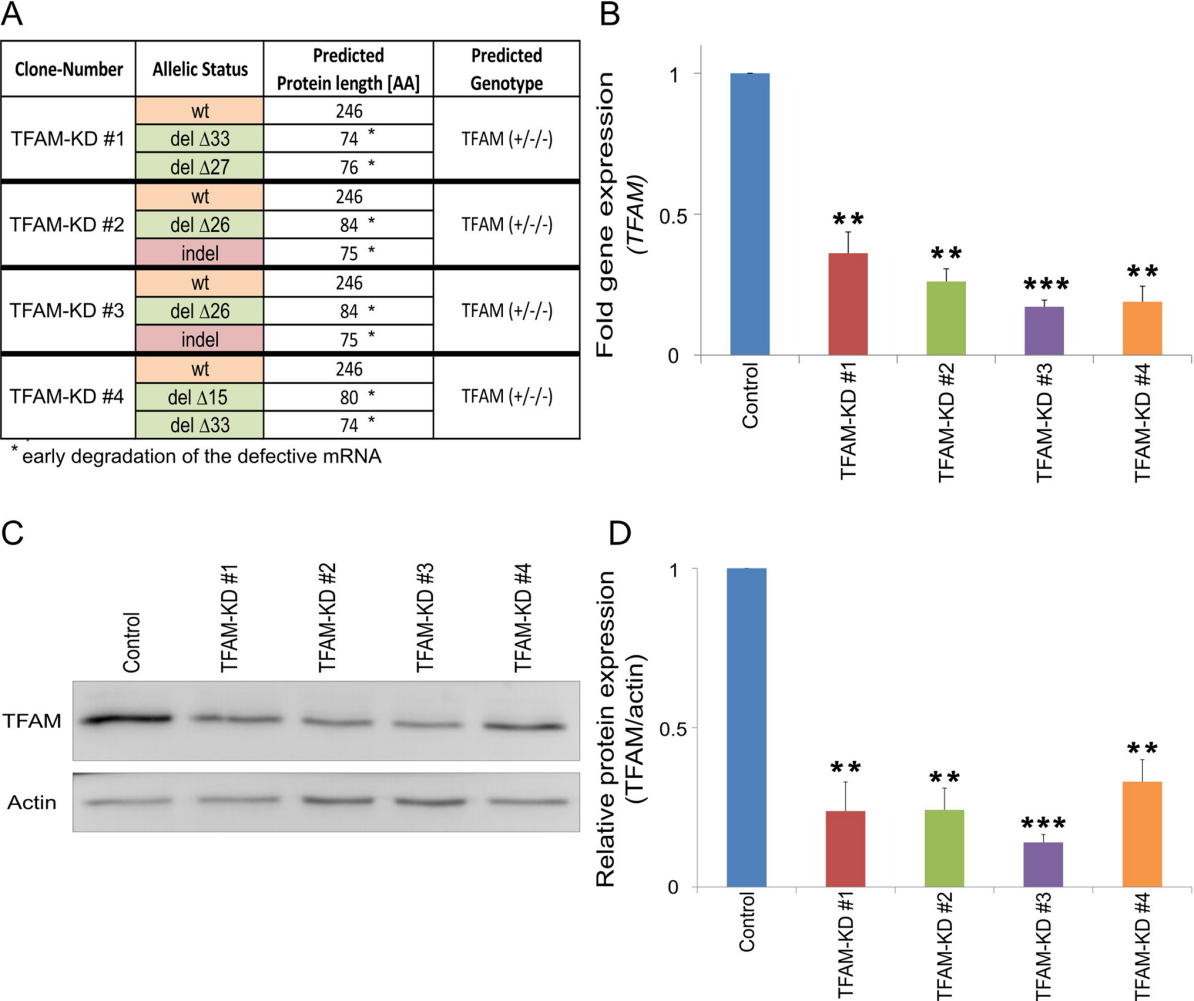
Validation of *TFAM* knockdown in *colorectal cancer* cells. After the introduction of deleterious mutations in *TFAM* using CRISPR-Cas9, four SW480 cell clones (TFAM-KD #1, TFAM-KD #2, TFAM-KD #3, TFAM-KD #4) were selected and *TFAM* mutations were confirmed through sequence analysis (**A**), changes in gene expression (**B**) and changes in protein expression (**C**, **D**). **A** Sequence analysis was used to identify the allelic status of cell clones and to predict the associated genotype. The TFAM protein length associated with this genotype was predicted with clustal omega. It should be noted that protein synthesis of mutated *TFAM* alleles is not expected to occur due to early degradation of defective mRNA. Sequencing of SW480 wild-type was used as a control (wt = wild-type, del = deletion, indel = insertion and deletion). **B** Gene expression of *TFAM* was measured by qPCR analysis and normalized to the reference gene *GAPDH*. Data from three experiments are shown and values represent means ± SEM (**p ≤ 0.01, ***p ≤ 0.001; paired t-test (two-tailed)). **C**,** D** Protein level of TFAM was measured and quantified by western blot analysis. 30 µg protein was loaded per lane and actin was used as a loading control. Values are means ± SEM (n = 3, **p ≤ 0.01, ***p ≤ 0.001; paired t-test (two-tailed))

To study the functional role of TFAM on tumorigenic properties of colorectal cancer cells, *TFAM* expression was manipulated in the cell line SW480 using CRISPR-Cas9. The selected gRNA of the CRISPR-Cas9 approach encompassed the exon–intron junction of exon 2 of the *TFAM* gene. All identified deleterious mutations consisted of deletions and indels disrupting this boundary, which resulted in a truncated and non-functional form of the protein (Fig. 1A). When we screened for TFAM-deficient cell clones, we only identified clones with at least one remaining wild-type allele. Because the colorectal cancer cell line SW480 displays a triploid karyotype [25], we were only able to isolate clones with two disrupted TFAM alleles and one wild-type allele (Fig. 1A). Based on this observation one might speculate that complete *TFAM* knockout is potentially lethal or at least disadvantageous for SW480 cells. This is consistent with the findings that a homozygote TFAM knock-out is embryonically lethal in mice [26, 27]. Next, we investigated mRNA and protein levels of TFAM by qPCR analysis and immunoblotting (Fig. 1B–D). All selected cell clones (TFAM-Knock-down (TFAM-KD) #1, TFAM-KD #2, TFAM-KD #3, TFAM-KD #4) exhibited a reduction in *TFAM* mRNA by 60–80% (p ≤ 0.01) in comparison to control cells (Fig. 1B). Moreover, the protein levels of TFAM were also reduced by 70–85% (p ≤ 0.01) compared to the control (Fig. 1C + D).

### TFAM deficiency leads to reduced mtDNA copy number and reduced expression of mtDNA-encoded genes

**Fig. 2:**
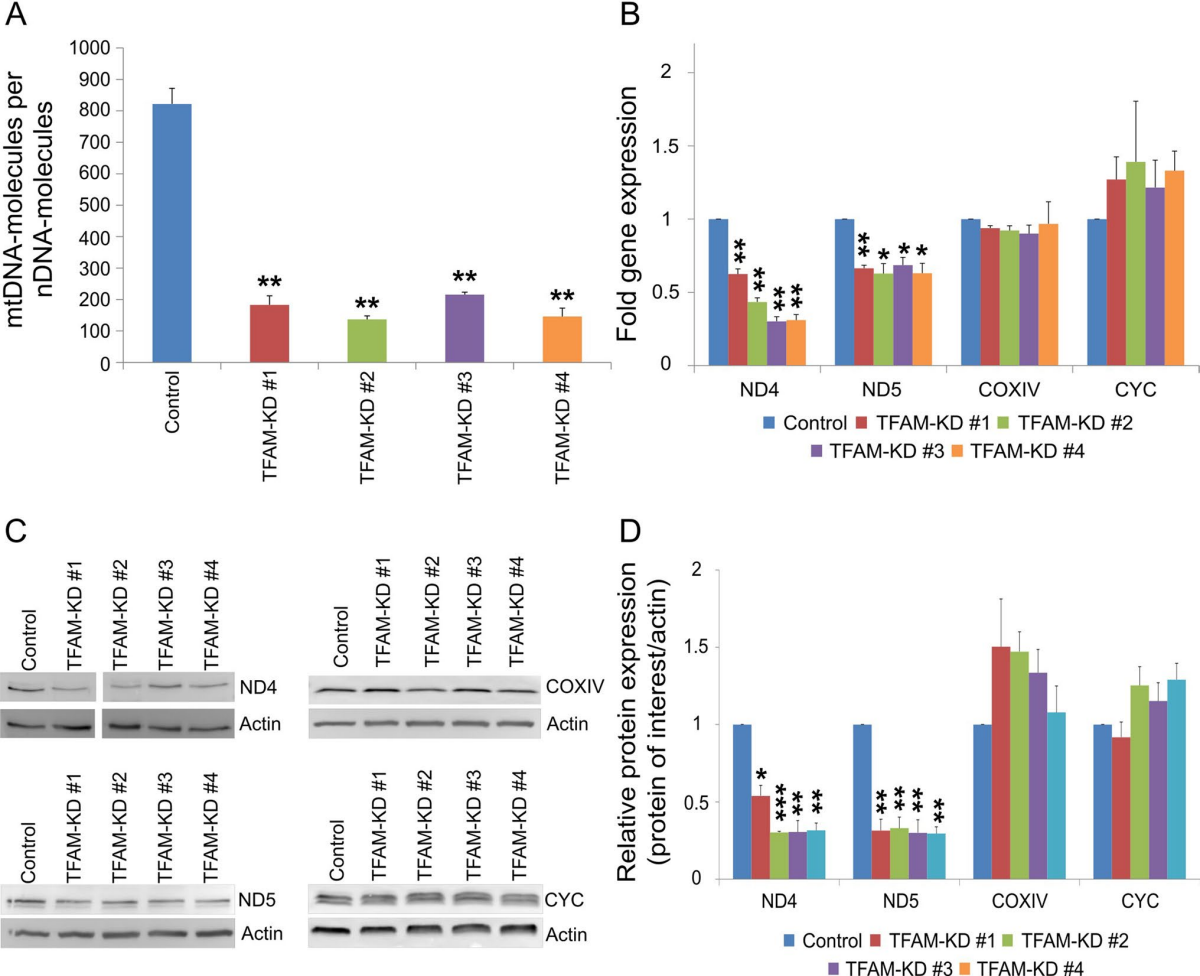
TFAM impacts mtDNA copy number and expression of mtDNA-encoded gens. **A** Quantification of relative mtDNA copy number by qPCR analysis (the mitochondrial loci *ND1* and *DLoop* in relation to nuclear loci *GAPDH* and *B2M*). Data are represented as means ± SEM (n = 3, **p ≤ 0.01; paired t-test (two-tailed)). **B** Quantification of gene expression of mitochondria-related genes. Expression of two nuclear-encoded genes (*COXIV, CYC*) and two mtDNA-encoded gens (*ND4, ND5*) were analyzed by qPCR analysis and normalized to the reference gene *GAPDH*. Data were shown from three experiments and values represent means ± SEM (*p ≤ 0.05, **p ≤ 0.01; paired t-test (two-tailed)). **C** Representative western blot images of analyzed mitochondrial proteins. 30 µg protein was loaded per lane and actin was used as a loading control. There was an irrelevant sample loaded on the ND4 blot and therefore the blot was cut between sample TFAM-KD #1 and #2. All original, non-cleaved Western Blot images are shown in the supplementary figure S3. **D)** Quantification of protein level of mitochondria-related genes. Two nuclear-encoded genes (*COXIV, CYC*) and two mtDNA-encoded gens (*ND4, ND5*) were tested. Data were shown from three experiments and values represent means ± SEM (*p ≤ 0.05, **p ≤ 0.01, ***p ≤ 0.001; paired t-test (two-tailed))

TFAM has been described as a key regulator of mtDNA replication and mtDNA expression [8]. Thus, we analyzed the impact of *TFAM* deficiency on mtDNA copy number and on the expression of mitochondrial genes. We performed qPCR analysis of two mtDNA loci (*ND1, D-Loop*) and two nuclear loci (*GAPDH, B2M*) to asses relative mtDNA levels in wild-type (control) and *TFAM*-deficient SW480 cells. *TFAM* deficiency resulted in an overall decrease of mtDNA copy number (137–216 copies) compared to control (822 copies) (p ≤ 0.01; Fig. 2A). In order to determine if these changes influence the expression of mtDNA encoded genes, qPCR analysis was performed of two mtDNA-encoded (*ND4, ND5*) and two nuclear-encoded (*CYC, COXIV*) genes, which all encode factors of the OXPHOS complex (Fig. 2B). While the expression of the mtDNA-encoded genes *ND4* and *ND5* was significantly decreased (p ≤ 0.05), *TFAM*-deficiency had no impact on the expression of the nuclear-encoded genes *CYC* and *COXIV* (p ≥ 0.05; Fig. 2B). As determined by immunoblot analyses, the same effects could be observed for the protein levels of these targets (Fig. 2C, D). While the protein levels of ND4 and ND5 were significantly decreased (p ≤ 0.05) in *TFAM*-deficient cells, the protein levels of the nuclear-encoded targets COXIV and CYC were not affected by TFAM (p ≥ 0.05; Fig. 2D). All original, non-cleaved Western Blot images are shown in the supplementary figure S3.

### TFAM deficiency inhibits human colorectal cancer cell growth

**Fig. 3:**
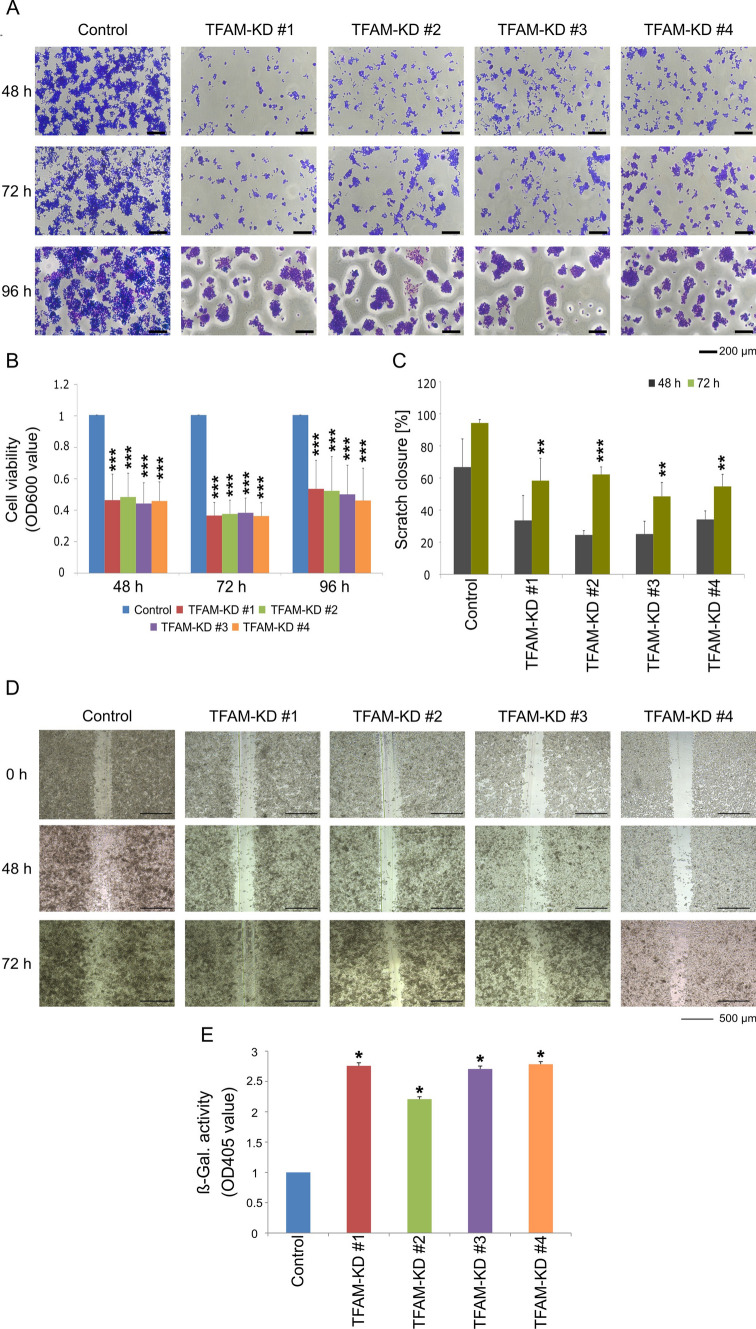
Knockdown of TFAM leads to reduced viability and proliferation of colorectal cancer cells. **A**, **B** SW480 control cells and *TFAM*-deficient cell clones were seeded in 96-well plates and crystal violet staining was quantitatively determined for optical absorbance after 48, 72 and 96 h. Values are means ± SEM (n = 3, ***p ≤ 0.001; paired t-test (two-tailed)). **C**,** D** Confluent monolayers of cells were scratched using a pipette tip and the closure of the scratch was measured at three time points (0, 48 and 72 h). All experiments were performed in triplicates. Values represent means ± SEM (**p ≤ 0.01, ***p ≤ 0.001 paired t-test (two-tailed)). **E** Measurement of senescence-associated β-galactosidase activity. The β-galactosidase activity was measured by absorbance at 405 nm and absorbance of control cells was normalized to one. Representative data were shown from three independent experiments and values represent means ± SEM (*p ≤ 0.05; paired t-test (two-tailed))

Next we were interested in functional consequences of *TFAM* downregulation in colorectal cancer cells. We compared cell viability, proliferation, apoptosis, senescence and ROS production in control and *TFAM*-deficient cells. Viability of *TFAM*-deficient cells was measured by crystal violet (Fig. 3A, B). Our results show that downregulation of *TFAM* expression leads to a very prominent (above 50%) decrease of cell viability at all tested conditions (p ≤ 0.001; Fig. 3A, B). Moreover, reduced *TFAM* expression led to delayed gap closure in scratch assay: 50–62% closure in *TFAM*-depleted cells compared to 94% scratch closure in control cells (p ≤ 0.01, Fig. 3C, D). However, the observed reduction in gap closure is most likely due to the effect of TFAM on cell proliferation. Previous studies indicate that *TFAM* deficiency may lead to increased production of ROS or apoptosis induction in cancer cells [12, 28]. Here, we did not detect any changes in ROS production or in the expression of apoptosis markers (Supplementary Figure S1 and S2). However, we observed higher activity of the senescence-associated enzyme β-galactosidase in *TFAM*-deficient cells (p ≤ 0.05, Fig. 3E).

### TFAM deficiency affects multiple signaling pathways in human colorectal cancer cells

**Fig. 4:**
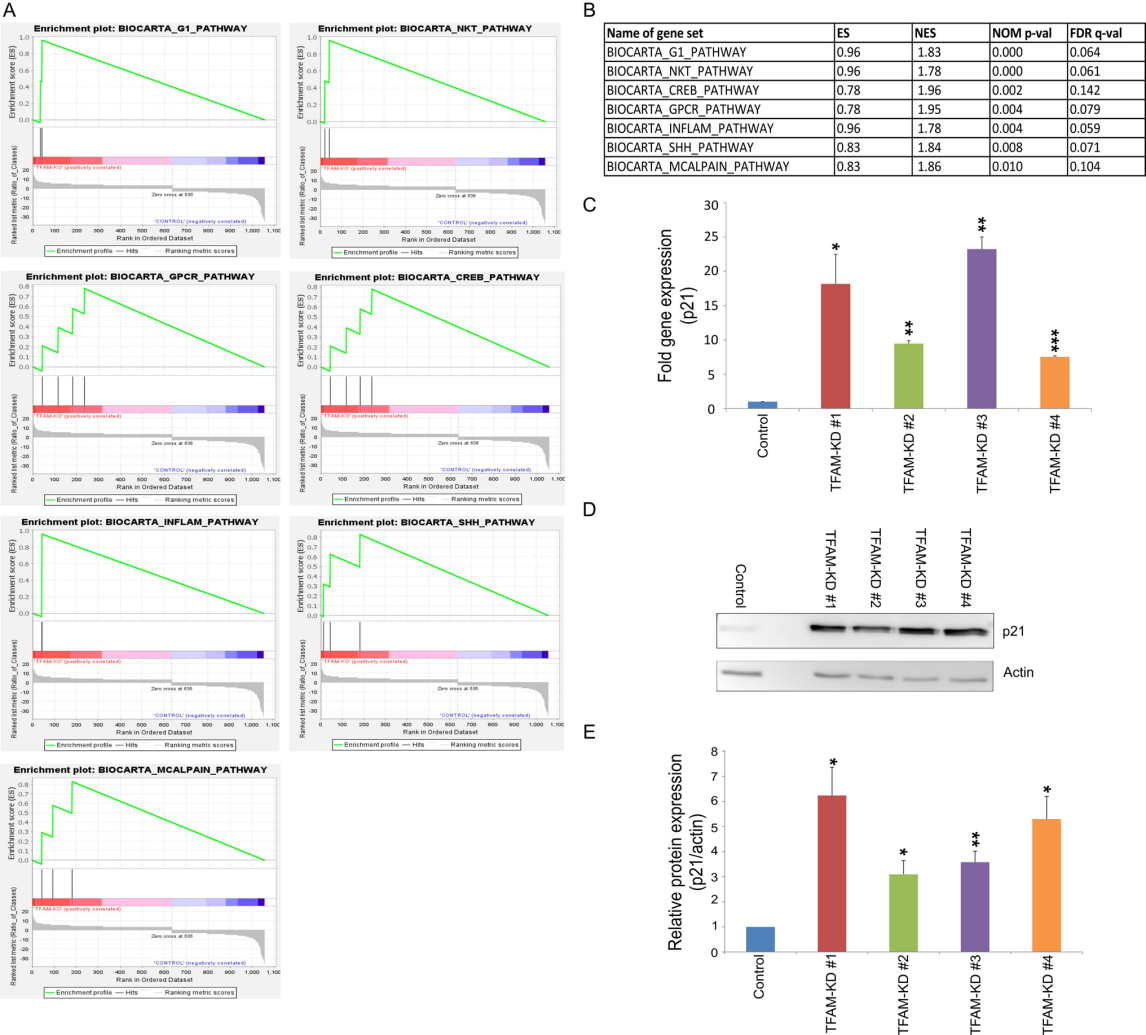
TFAM impacts the expression of *CDKN1A/p21*. **A**, **B** Gene sets enriched in *TFAM*-deficient cells. Enrichment score (ES) reflects the degree to which a gene set is overrepresented at the top or bottom of a ranked list of genes. Normalized enrichment score (NES) represents enrichment score normalized across analyzed gene sets. Statistical significance is calculated by nominal p-value of the ES using an empirical phenotype-based permutation test. Gene sets from the MSigDB database were tested and gene sets with a false discovery rate (FDR) < 0.25 and nominal p-value < 0.01 were considered significantly enriched in a prior defined set of genes. **C** Gene expression of *CDKN1A/p21* was measured by qPCR analysis and normalized to the reference gene *GAPDH*. Data were shown from three experiments and values represent means ± SEM (*p ≤ 0.05; **p ≤ 0.01, ***p ≤ 0.001; paired t-test (two-tailed)). **D**, **E** Western blot analysis and quantification of p21. 25 μg protein was loaded per lane and actin was used as a loading control. Values are means ± SEM (n = 3, *p ≤ 0.05; **p ≤ 0.01, paired t-test (two-tailed))

In order to dissect the molecular alterations induced by downregulation of *TFAM*, RNA sequencing was performed on *TFAM*-deficient and control SW480 cells. Differential gene analysis (padj ≤ 0.05; differentially expressed genes in Supplementary Table S5) revealed 637 upregulated and 421 downregulated genes in *TFAM*-deficient cells. As expected, the expression of *TFAM* was significantly reduced in the generated *TFAM*-deficient cell clones in comparison to the control (stat = -19.61; Supplementary Table S5). In total, 7 out of 203 BioCarta pathways were significantly enriched in *TFAM*-deficient compared to control colorectal cancer cells (false discovery rate (FDR) < 0.25; nominal p-value < 0.01). The BioCarta signaling pathways enriched in *TFAM*-deficient colorectal cancer cells included G1/S check point regulation, selective expression of chemokine receptors during T-cell polarization (Natural killer T (NKT) cells), transcription factor CRE-binding protein (CREB) signaling, signaling pathway from G-protein families, cytokines and inflammatory response, sonic hedgehog (shh) signaling, and M-calpain pathway (Fig. 4A, B).

### *TFAM* deficiency leads to increased expression of *CDKN1A/p21*

Based on functional assays showing a major effect of *TFAM* deficiency on cellular senescence and cell growth, we were searching for molecular targets of TFAM that could mediate these effects. Interestingly, gene set enrichment analysis (GSEA) identified the G1/S check point pathway, which included the genes *cyclin dependent kinase inhibitor 1A (CDKN1A)* and *transforming growth factor beta 2 (TGFB2)*, as the most significantly enriched gene set in *TFAM*-deficient cells (Fig. 4A, B). Moreover, *CDKN1A* was among the top highly significant upregulated genes in *TFAM*-deficient cells. *CDKN1A* encodes p21, a cell cycle regulating protein, that plays a key role in inhibiting cell division through the inhibition of cyclin-dependent kinases [29]. Using quantitative PCR we could confirm that the expression of *CDKN1A/p21* was 8–25 fold-changes higher in *TFAM*-deficient colorectal cancer cells when compared to the control (p ≤ 0.05; Fig. 4C). Furthermore, protein levels of p21 were increased by 3–6 times in *TFAM*-deficient cells (p ≤ 0.05, Fig. 4D, E). These findings align with our observation regarding decreased cell proliferation and increased senescence in *TFAM*-deficient cells, since overexpression of *CDKN1A/p21* is classically correlated with cellular senescence [30, 31]. Due to its role as a key regulator of cell cycle that initiates cell cycle arrest in response to DNA damage, p21 has an impact on the proliferation of different cancer cell types [30, 32, 33].

## Discussion

Alterations in mtDNA copy number have been investigated across various tumor entities revealing a complex landscape. For example, studies in gastric cancer, breast cancer and non-small cell lung cancer revealed decreased mtDNA levels [34–36], while in prostate tumors and esophageal squamous cell cancer mtDNA copy numbers were increased [37, 38]. In colorectal cancer both, an increase and a decrease in mtDNA copy numbers, were observed [39, 40]. Interestingly, high mtDNA levels were associated with poor outcome and a higher risk of metastases for patients, making the exploration of mtDNA copy number regulation an intriguing topic in the context of tumorigenic properties of colorectal cancer [5].

In the present study we found that *TFAM* deficiency leads to decreased mtDNA levels in the colorectal cancer cell line SW480 and reduced expression of mtDNA-encoded and OXPHOS-related genes. SW480 is one of the most frequently studied cancer cell lines and represents a common colorectal cancer type which is characterized as microsatellite stable and by deleterious TP53 mutations and activating KRAS mutations [15]. In agreement with our findings, previous studies have reported in various murine cells and zebrafish that reduction of TFAM expression leads to ~ 50% decrease in mtDNA levels showing that TFAM acts as a limiting determinant for the mtDNA copy number [11, 41–44].

Interestingly, a study from Sun et al*.* showed that changes of mtDNA levels, mediated by TFAM, impact cancer progression in microsatellite-stable (MSS) colorectal cancer cells [45]. They found that increased mtDNA levels facilitated cell proliferation and metastasis of colorectal cancer cells indicating that mtDNA may have a tumor-promoting role [45]. In our study we did not find significant evidence by using the TCGA database that downregulation of TFAM in colorectal cancers is associated with better survival (data not shown). However, in agreement with Sun et al. [45], we showed that decreased mtDNA copy numbers in *TFAM*-deficient colorectal cancer cells diminish tumorigenic properties by reducing cell proliferation. While Sun et al*.* considered these observations in the context of oxidative phosphorylation [45], our goal was to identify potential targets that link *TFAM* deficiency and reduced cell proliferation. Interestingly, total mRNA expression profiling revealed upregulation of the cell cycle inhibitor *CDKN1A/p21* in *TFAM*-deficient colorectal cancer cells. It has been shown that p21 is a possible target of TFAM, as disruption of *TFAM* in mouse skin fibroblasts and non-small cell lung cancer cells has led to elevated expression of *p21* in previous studies [12, 46].

The p21 protein is a critical regulator of different tumorigenesis related processes including cell cycle, cellular senescence and apoptosis in response to cellular stress [47]. It has been described as a tumor-suppressor and a tumor-promoting factor, whereby the tumorigenic potential of p21 is attributed to its anti-apoptotic activity [48, 49]. The tumor-suppressive function is based on its ability to inhibit the cell cycle and promote cellular senescence [50–53]. In our study, p21 upregulation was not only correlated with reduced cellular proliferation in colorectal cancer cells, it also appeared with upregulation of senescence associated β-galactosidase. In that way *TFAM* deficiency promotes the tumor-suppressive functions of the p21 protein.

TFAM and p21 have previously been linked by a process called retrograde signaling [54]. Retrograde signaling is described as a biological process where signaling molecules are released from organelles such as mitochondria, taking an impact on the transcription of nuclear-encoded genes [55]. It has been shown that the absence of mtDNA copy number in osteosarcoma cells initiates a retrograde mechanism that results in phosphorylation of the CRE-binding (CREB) protein [54]. The CREB transcription factor then leads to *TP53*-mediated overexpression of *CDKN1A/p21* resulting in reduced tumorigenesis of the cells [54]. It is noteworthy that this scenario is speculative and other mechanisms must exist how TFAM induces *CDKN1A/p21* upregulation. In the present study we used SW480 cells that harbor deleterious TP53 mutations. This suggests a TP53-independent mechanism for *CDKN1A/p21* upregulation in those cells*.* Interestingly, a study from Wang et al*.* showed that the induction of p21 in CRC cell lines is quite high and robust, particularly in those lacking p53, underlying this observation [56].

There is a potential link between mitochondrial dysfunction, specifically involving TFAM, and the p53-independent activation of p21. Loss of TFAM and depletion of mtDNA can disrupt mitochondrial function, leading to cellular stress that might trigger the upregulation of p21 through p53-independent pathways [12]. As we did not observe any changes in the ROS-production, this pathway does not seem to be relevant for our findings. Another aspect to consider is the role of MELK (Maternal Embryonic Leucine Zipper Kinase) [57]. MELK is a cell cycle-regulated kinase that has been implicated in various cellular stress responses and can influence p21 expression [57]. It is possible that MELK could be involved in the p53-independent regulation of p21 in the context of mitochondrial dysfunction. MELK's activity might intersect with pathways that are altered by TFAM loss, further contributing to p21 upregulation. Additionally, mitochondrial dysfunction can affect cellular energy metabolism, leading to activation of pathways like AMPK (AMP-activated protein kinase). AMPK is a key regulator of cellular energy homeostasis and can modulate p21 expression through p53-independent routes, especially in response to energetic stress [58]. This suggests that disruptions in mitochondrial function caused by TFAM loss might contribute to p21 upregulation via mechanisms related to altered metabolic signaling rather than ROS production.

In summary, our study reveals that *TFAM* deficiency in colorectal cancer cells leads to a decrease in mtDNA levels, accompanied by downregulated expression of mtDNA-encoded and oxidative phosphorylation-related genes. This reduction in mtDNA copy number correlates with inhibited cell proliferation and an upregulation of the tumor-suppressive protein p21 suggesting a potential link between TFAM, mtDNA levels and the p21-mediated tumor-suppressive pathway. While the exact mechanism remains unclear, our findings provide insights into the intricate regulation of mtDNA in colorectal cancer and underscore the significance of TFAM in modulating tumorigenic properties. In future, it might be interesting to explore the consequences of TFAM expression in colorectal cancer samples on clinicopathological features.

## Supplementary Information

Additional file 1.

Additional file 2.

## Data Availability

The datasets generated and/or analyzed in the present study are available from the corresponding author on reasonable request.

## Abbreviations

AMPK: AMP-activated protein kinase
B2M: Beta-2-Microglobulin
CDKN1A: Cyclin-dependent kinase inhibitor 1A (corresponding protein: p21)
cDNA: Complementary DNA
COXIV: Cytochrome-c-Oxidase-Subunit 4
CREB: CRE-binding protein
ct: Threshold cycle number
CYC: Cytochrome C
FACS: Fluorescence-activated cell sorting
FBS: Fetal bovine serum
FDR: False Discovery Rate
GAPDH: Glyceraldehyde 3-phosphate dehydrogenase
GFP: Green fluorescent protein
gRNA: Guide-RNA
HMG box: High mobility group box
KD: Knock-down
MELK: Maternal Embryonic Leucine Zipper Kinase
MSS: Microsatellite-stable
mtDNA: Mitochondrial DNA
ND1: NADH dehydrogenase 1
ND4: NADH dehydrogenase 4
ND5: NADH dehydrogenase 5
NKT: Natural killer T
OXPHOS: Oxidative phosphorylation system
padj: Adjusted *p*-value
qPCR: Quantitative real-time PCR
RNA: Ribonucleic acid
shh: Sonic hedgehog
TFAM: Transcription factor A, mitochondrial
TGFB2: Transforming growth factor beta 2
wt: Wild-type
