# Supplementary material for: Decreased mitochondrial transcription factor A and mitochondrial DNA copy number promote cyclin-dependent kinase inhibitor 1A expression and reduce tumorigenic properties of colorectal cancer cells
Source: Discov Oncol. 2024 Nov 24;15:701. doi: 10.1007/s12672-024-01538-4 (PMC11586319; doi:10.1007/s12672-024-01538-4)
Supplement: Supplementary file 1 — Additional file 1. [file 12672_2024_1538_MOESM1_ESM.docx]

**Supplementary figures**


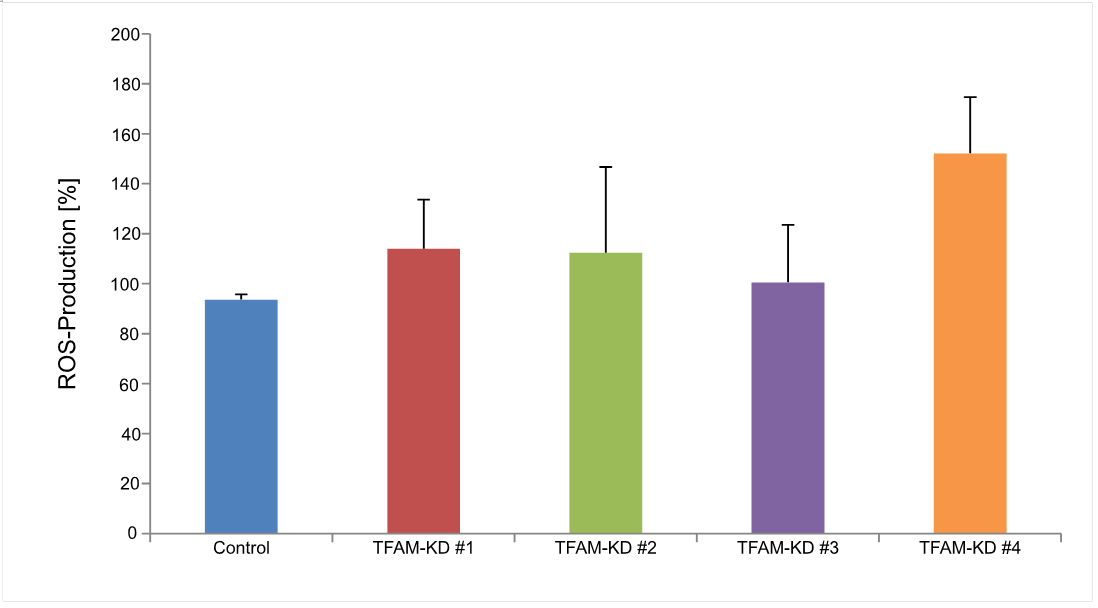


**Figure S1: Measurement of ROS-production.** The production of ROS was assessed by measuring fluorescence intensity (515 – 545 nm) following cell treatment with DCFH-DA. The graph is based on three biological replicates, each incorporating the median of the measured fluorescence intensity. The bars represent the mean of biological replicates (n = 3) ± SD. A two-sided unpaired t-test was conducted for each cell clone compared to the wild type (p > 0.05 = non-significant).


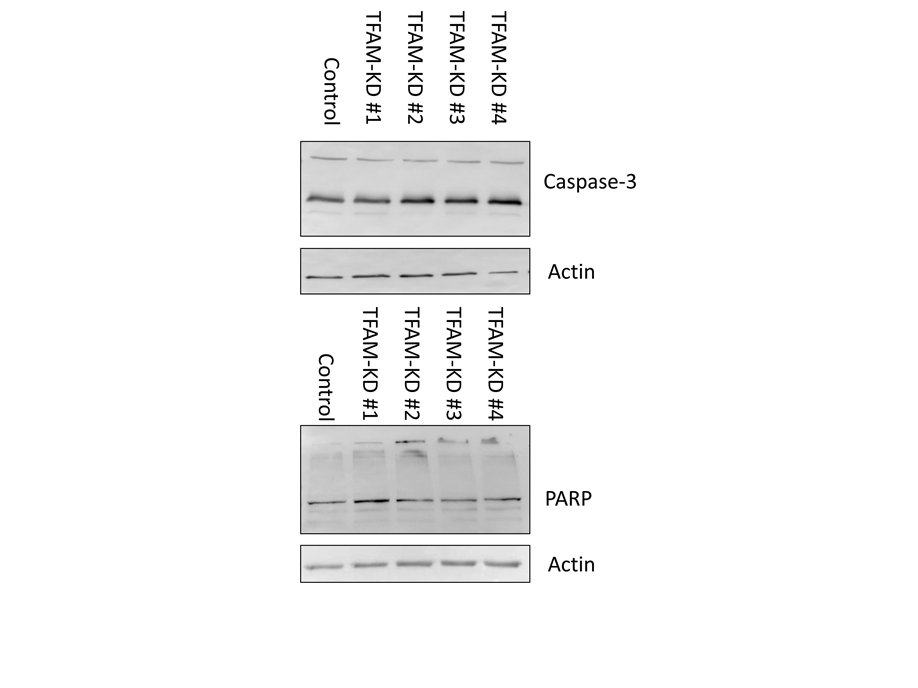


**Figure S2: Immunoblot analysis of apoptosis-related proteins.** For apoptosis detection, immunoblot analysis for full-length and cleaved caspase-3 as well as PARP was performed. Actin served as loading control. 30 μg protein lysates were separated using an SDS-PAGE gel.


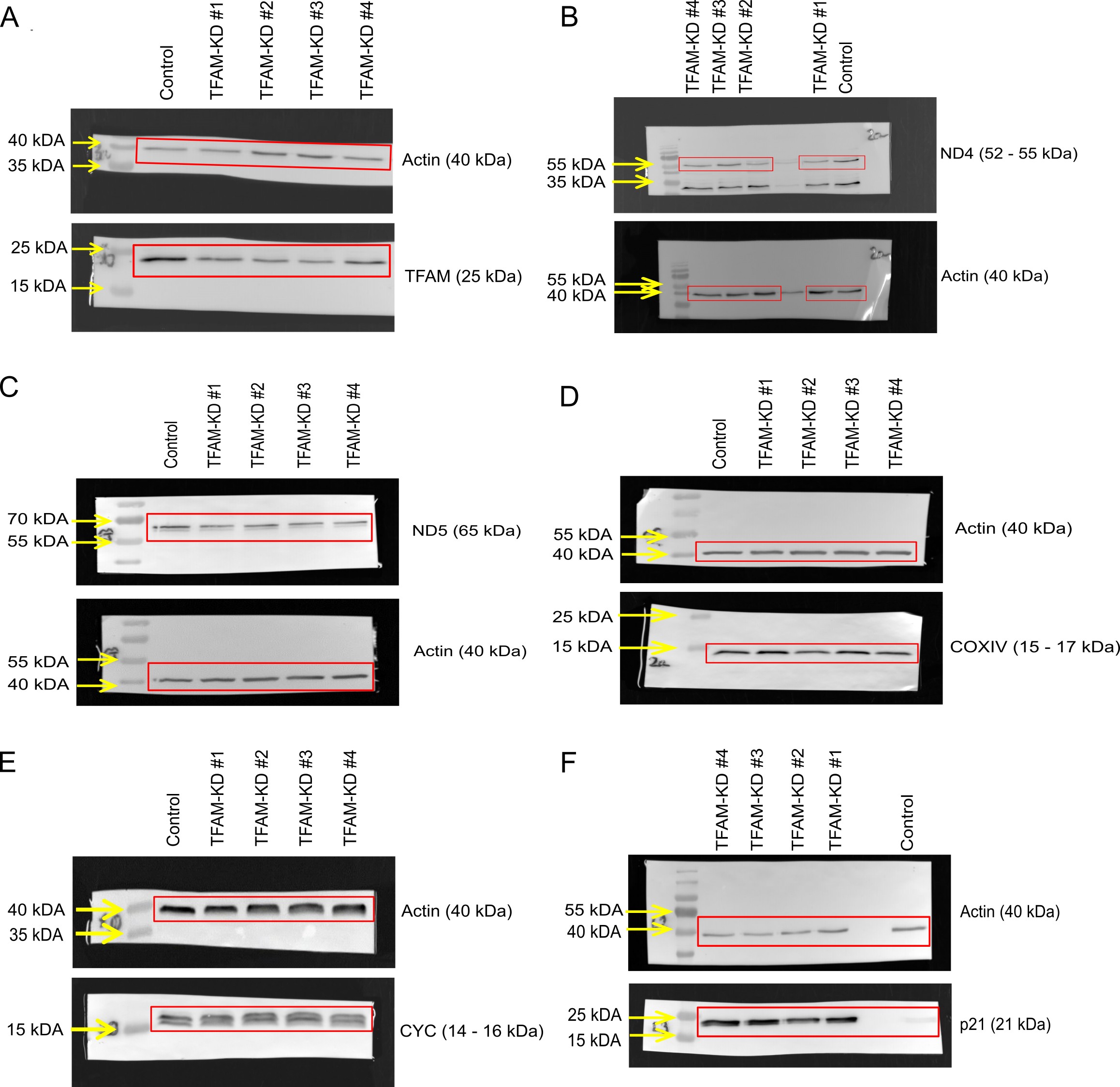


**Figure S3: Original, non-cleaved Western Blot images.** The red boxes indicate the regions of the original blots used for the main figures. (A) The image shows the original Western Blot for the detection of TFAM in the main figure 1C. (B) The image shows the original Western Blot for the detection of ND4 in the main figure 2C. One sample was excluded from the main figure. To address this, we have added a comment in the figure legend to clarify the blot’s cropping for the reader. The ND4 signal is in accordance with the signal obtained from the manufacturer for this particular antibody. Therefore, multiple bands were expected for ND4 in the western blot, whereby the band at 52 to 55 kDa was clearly identified by the manufacturer as the desired protein band. Due to secondary antibodies from different host species, Actin (mouse) and ND4 (rabbit) could be detected on the same membrane without any signal interferences. For a better understanding, the Western Blot image has been mirrored in the main figure 2C. (C) The image shows the original Western Blot for the detection of ND5 in the main figure 2C. Due to secondary antibodies from different host species, Actin (mouse) and ND5 (rabbit) could be detected on the same membrane without any signal interferences. For a better understanding, the Western Blot image has been mirrored in the main figure 2C. (D) The image shows the original Western Blot for the detection of COXIV in the main figure 2C. (E) The image shows the original Western Blot for the detection of CYC in the main figure 2C. (F) The image shows the original Western Blot for the detection of p21 in the main figure 4D. For a better understanding, the Western Blot image has been mirrored in the main figure 4D.
